# Cutaneous macroglobulinosis with Waldenström macroglobulinemia and Bing-Neel syndrome: A case report

**DOI:** 10.1016/j.jdcr.2025.04.009

**Published:** 2025-04-25

**Authors:** Amir Khogeer, Alain Dupuy

**Affiliations:** Department of Dermatology, Rennes University Hospital, Rennes, France

**Keywords:** Bing-Neel syndrome, case report, cutaneous macroglobulinosis, lymphoplasmacytic lymphoma, Waldenström macroglobulinemia

## Introduction

Waldenström macroglobulinemia (WM) is a lymphoproliferative neoplasm of B cells characterized by small lymphocytes and monoclonal IgM gammopathy.^1^ Specific cutaneous manifestations related to WM, collectively termed cutaneous macroglobulinosis (CM), result from neoplastic B-cell infiltration and monoclonal IgM deposition in the skin.^2^ Bing-Neel syndrome (BNS) is a rare neurological manifestation of WM characterized by lymphoplasmacytic infiltration into the leptomeningeal tissue and/or the central nervous system.^3^ More than 100 cases of BNS have been reported, including those documented by Simon et al (44 cases) and Ina Ly et al (36 cases).^4^^,^^5^

This article details the case of a 55-year-old male with CM secondary to WM, who later developed BNS. This is among the few documented cases of a patient presenting with WM, CM, and BNS simultaneously.

## Case report

**Fig 1:**
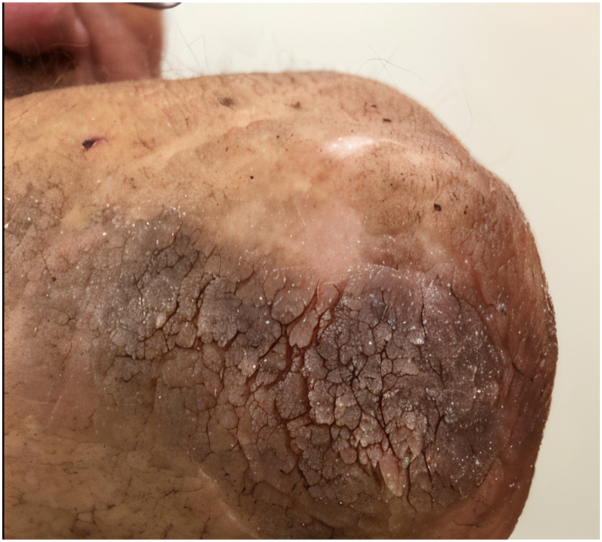
Flesh-colored, translucent papules grouped into plaque located on the elbow.

**Fig 2:**
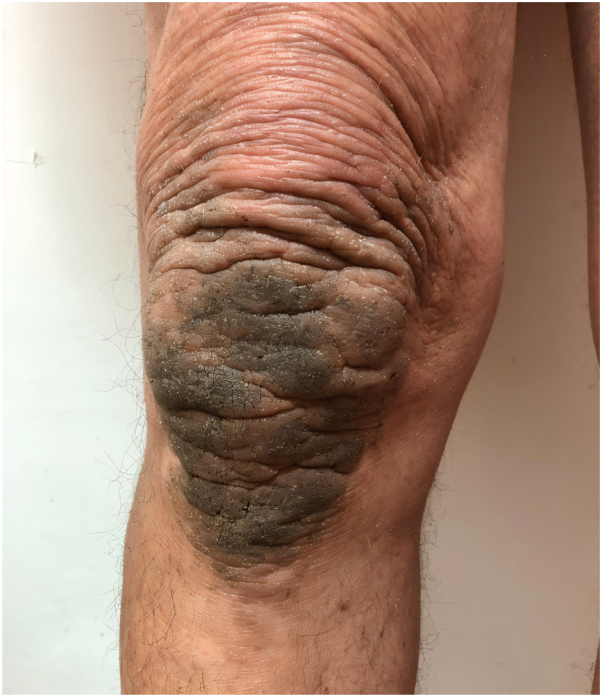
Dark, translucent papules grouped into plaque located on the knee.

A 55-year-old male presented with diffuse skin infiltration affecting the hands, forearms, arms, and neck, characterized by multiple flesh-colored and dark micropapules. Three years earlier, he had been diagnosed with anti-myelin-associated glycoprotein paraprotein neuropathy related to Waldenström macroglobulinemia following peripheral neuropathy in the lower limbs. Skin examination revealed flesh-colored to dark micropapules across the hands, forearms, arms, neck, knees, chest, and face, with no lymphadenopathy or hepatosplenomegaly (Figs 1 and 2).

Blood tests showed hemoglobin at 13.8 g/dL, platelets at 352 × 10^9^/L, and leukocytes at 11.3 × 10^9^/L. Serum electrophoresis revealed a monoclonal peak in the β2 region with significantly elevated IgM levels (33 g/L).

**Fig 3:**
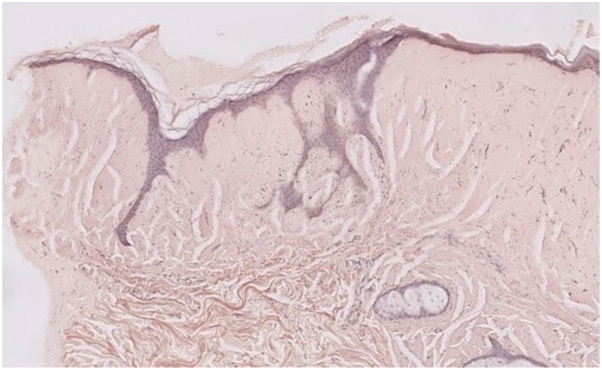
Histopathologic features of the skin biopsy showing positive Periodic Acid–Schiff staining with hyaline material in the dermis, perivascular lymphocytic infiltration, and neovascularization (H&E, ×200).

**Fig 4:**
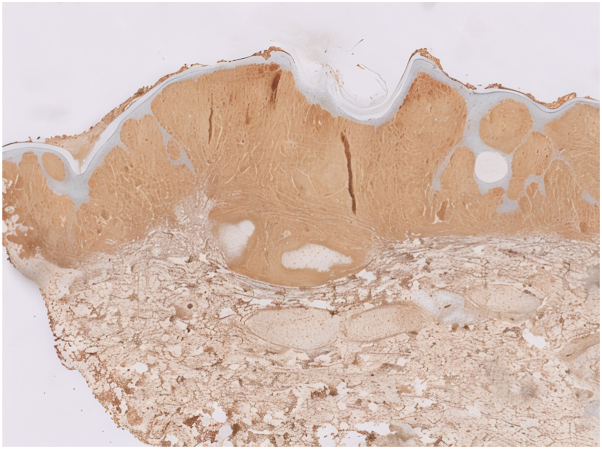
Immunohistochemical staining demonstrating strong positivity for IgM in the dermis (IgM IHC, ×200).

A skin biopsy revealed positive Periodic Acid–Schiff staining, negative Congo red staining, and a markedly thinned epidermis with ridge stretching. The dermis was filled with abundant hyaline material and demonstrated perivascular lymphocytic infiltration, neovascularization, and eosinophilic deposits (Fig 3). Immunohistochemical examination showed strong IgM positivity, confirming the diagnosis of CM associated with WM (Fig 4).

The patient was diagnosed with CM associated with WM and started on 2 cycles of RCD (rituximab, cyclophosphamide, and dexamethasone). He was referred to the oncology department, where treatment was initiated.

After the first 2 cycles, he reported progressive cognitive, memory, hearing, and visual impairments. BNS was suspected. A magnetic resonance imaging scan with gadolinium revealed no morphological abnormalities or suspicious contrast enhancement in the brain or meninges. A lumbar puncture with flow cytometry revealed lymphoplasmacytic cells with clonality, confirming the diagnosis of BNS. The patient’s treatment regimen was adjusted, replacing cyclophosphamide with fludarabine, given its superior meningeal penetration. He received 6 cycles of rituximab and intrathecal injections. Over time, the patient reported gradual improvements in cognitive, visual, and auditory functions, along with a progressive regression of cutaneous lesions. Six months post-therapy, laboratory evaluation showed serum IgM: 2.7 g/L and a normal lumbar puncture revealing rare nonatypical lymphocytes and no proteinorrhagia (0.71 g/L).

## Discussion

CM is a rare manifestation of WM, occurring in approximately 5% of cases.^2^ CM often mirrors IgM levels and disease activity. In this case, CM regressed with effective treatment for WM and BNS, supporting its role as a marker of disease progression.

BNS, a rare complication of WM, typically arises after WM diagnosis and has varied clinical presentations, often delaying diagnosis.^3^

According to the classification proposed by Ina Ly et al, BNS can be divided into 2 subtypes: type A, characterized by direct neoplastic infiltration of the CNS, and type B, associated with IgM deposition leading to immune-mediated damage. Based on the presence of clonal lymphoplasmacytic cells in the cerebrospinal fluid, our case is consistent with type A involvement.^5^

The diagnosis of BNS remains challenging, with some experts considering a histological biopsy of the cerebrum or meninges as the gold standard, though cerebrospinal fluid flow cytometry remains a key diagnostic tool.^6^

## Conclusion

This case underscores the complexities of WM when accompanied by CM and BNS. CM can serve as a marker of WM activity, while BNS represents a severe neurological complication requiring early detection and specific treatment. The patient’s improvement following tailored therapy highlights the importance of a comprehensive approach in managing these interconnected conditions.

## Conflicts of interest

None disclosed.

## References

[1] Kaseb H., Gonzalez-Mosquera L.F., Parsi M. (2024).

[2] Gressier L., Hotz C., Lelièvre J.D. (2010). Cutaneous macroglobulinosis: a report of 2 cases. Arch Dermatol.

[3] Nanah A., Al Hadidi S. (2021). Bing-Neel syndrome: update on the diagnosis and treatment. Clin Lymphoma Myeloma Leuk.

[4] Simon L., Fitsiori A., Lemal R. (2015). Bing-Neel syndrome: clinical and imaging findings in 44 cases. Haematologica.

[5] Ly K.I., Fintelmann F.J., Forghani R. (2011). Bing-Neel syndrome: a multicenter retrospective study of 36 patients. Clin Lymphoma Myeloma Leuk.

[6] Minnema M.C., Kimby E., D’Sa S. (2009). Diagnosis and treatment of Bing-Neel syndrome: an evidence-based review. Haematologica.

